# Recruitment of Intratumoral CD103^+^ Dendritic Cells by a CXCR4 Antagonist-Armed Virotherapy Enhances Antitumor Immunity

**DOI:** 10.1016/j.omto.2019.06.003

**Published:** 2019-07-03

**Authors:** Anna Mistarz, Marcin P. Komorowski, Matthew A. Graczyk, Margaret Gil, Aimin Jiang, Mateusz Opyrchal, Hanna Rokita, Kunle O. Odunsi, Danuta Kozbor

**Affiliations:** 1 Department of Immunology, Roswell Park Comprehensive Cancer Center, Buffalo, NY 14263, USA; 2 Department of Medicine, Roswell Park Comprehensive Cancer Center, Buffalo, NY 14263, USA; 3 Faculty of Biochemistry, Biophysics, and Biotechnology, Jagiellonian University, Kraków, Poland; 4 Center for Immunotherapy, Roswell Park Comprehensive Cancer Center, Buffalo, NY 14263, USA; 5 Department of Gynecologic Oncology, Roswell Park Comprehensive Cancer Center, Buffalo, NY 14263, USA

**Keywords:** virotherapy, CXCR4 antagonist, cancer vaccine, dendritic cells, T cells

## Abstract

Intratumoral dendritic cells play an important role in stimulating cytotoxic T cells and driving antitumor immunity. Using a metastatic ovarian tumor model in syngeneic mice, we explored whether therapy with a CXCR4 antagonist-armed oncolytic vaccinia virus activates endogenous CD103^+^ dendritic cell responses associated with the induction of adaptive immunity against viral and tumor antigens. The overall goal of this study was to determine whether expansion of CD103^+^ dendritic cells by the virally delivered CXCR4 antagonist augments overall survival and *in situ* boosting with a tumor antigen peptide-based vaccine. We found that locoregional delivery of the CXCR4-A-armed virus reduced the tumor load and the immunosuppressive network in the tumor microenvironment, leading to infiltration of CD103^+^ dendritic cells that were capable of phagocytic clearance of cellular material from virally infected cancer cells. Further expansion of tumor-resident CD103^+^ DCs by injecting the FMS-related tyrosine kinase 3 ligand, the formative cytokine for CD103^+^ DCs, provided a platform for a booster immunization with the Wilms tumor antigen 1 peptide-based vaccine delivered intraperitoneally with polyriboinosinic:polyribocytidylic acid as an adjuvant. The vaccine-induced antitumor responses inhibited tumor growth and increased overall survival, indicating that expansion of intratumoral CD103^+^ dendritic cells by CXCR4-A-armed oncovirotherapy treatment can potentiate *in situ* cancer vaccine boosting.

## Introduction

To be effective, cancer vaccine strategies need to promote the release of tumor antigens in the context of immunogenic tumor cell death (ICD), limit multiple levels of immunosuppression in the tumor microenvironment (TME), and increase intratumoral dendritic cell (DC) populations capable of stimulating cytotoxic T cells and driving immune responses against cancer.^1^, ^2^ Alongside traditional ICD inducers like selected chemotherapies and radiation (reviewed in Galluzzi et al.^3^), oncolytic viruses (OVs) have emerged as new members of this class of agents.^4^ Oncolytic virotherapy has been recognized as a form of immunotherapy, with a herpes simplex virus expressing granulocyte-macrophage colony-stimulating factor (GM-CSF) recently approved by the Food and Drug Administration^5^ and other vectors, including vaccinia virus, undergoing extensive evaluation in multiple preclinical and clinical trials.^6^, ^7^, ^8^, ^9^, ^10^, ^11^ Although OVs have shown limited clinical efficacy as a monotherapy, emerging data suggest that combination with conventional ICD-inducing chemotherapeutic agents,^8^ checkpoint inhibitors to combat PD-1/PDL-1-mediated immune suppression,^12^, ^13^, ^14^ and adjuvanted vaccines^15^ holds considerable promise. We have recently demonstrated that the innate resistance properties of highly metastatic ovarian tumors, together with the tumor immunosuppressive network, could be overcome by the oncolytic vaccinia virus (OVV)-delivered CXCR4 antagonist (CXCR4-A), which was particularly effective in combination with doxorubicin-mediated killing.^8^ Because the CXCL12/CXCR4 axis plays multiple pleiotropic roles in the progression of ovarian cancer, including stimulation of vascular endothelial growth factor (VEGF)-mediated angiogenesis,^16^ intratumoral recruitment of endothelial progenitor cells,^17^ as well as accumulation of CD11b^+^Gr1^+^ myeloid-derived suppressor cells (MDSCs)^18^ and T regulatory cells (Tregs),^19^ modulation of this axis affects innate and adaptive immune mechanisms of tumor destruction by increasing T lymphocyte infiltration as well as recently reported responses to checkpoint blockers.^20^ Therefore, modulation of the CXCL12/CXCR4 axis in ovarian cancer could affect multiple aspects of tumor pathogenesis, including immune dysregulation.

Several CXCR4 antagonists have demonstrated antitumor efficacy in preclinical models and have been evaluated in early clinical trials.^21^, ^22^, ^23^, ^24^ However, given the abundant expression of CXCR4 by many cell types, including those of the CNS and gastrointestinal and immune systems,^25^ the side effects of these antagonists need to be taken into consideration. Furthermore, the effect of soluble CXCR4 antagonists on the mobilization of CXCR4-expressing bone marrow (BM)-derived stem and progenitor cells represents an additional concern, particularly when combined with chemotherapeutic agents, because of the potential for adverse effects on hematopoiesis.^26^, ^27^ The potential effect of delivering a CXCR4-A “payload” by OVV may also depend on the route of administration of the armed virus, affecting both intratumoral viral titers and accumulation of CXCR4-A at the tumor site or in systemic tissues. This may affect the recruitment of immune cells, including the CD103^+^ DCs or classical type 1 DCs (cDC1s), which excel in priming and cross-presentation of tumor antigens to CD8^+^ T cells, and CD11b^+^ DCs or cDC2s, which are more potent at driving CD4^+^ helper T cell responses.^28^ Accumulating evidence suggests that tumor lesions enriched in type I interferon (IFN)-induced genes are also rich in T cells and that type I IFN production by the CD103^+^ DC lineage controls spontaneous T cell priming to tumor antigens.^29^ On the other hand, defective recruitment and activation of CD103^+^ DCs leads to reduced cross-priming of CD8^+^ T cells and poorly infiltrated or “cold” tumors.^30^, ^31^ Thus, increased myeloid cell commitment to the CD103^+^ DC lineage and activation of intratumoral CD103^+^ DCs could substantially enhance the effector phase of antitumor T cell responses.

Understanding the mechanisms that regulate the abundance of tumor-infiltrating lymphocytes (TILs) in the TME could unveil new therapeutic mechanisms. Because intratumoral DCs are necessary for enhanced T cell tumor responses,^2^, ^32^ we investigated the effect of the armed oncolytic virotherapy (OVV-CXCR4-A) used alone or in combination with the growth factor FMS-related tyrosine kinase 3 ligand (FLT3L; referred to hereafter as FL) on mobilization of infiltration of CD103^+^ and CD11b^+^ DCs to the tumor site and induction of T cell tumor responses. Using an intraperitoneal ovarian tumor model (ID8-T) enriched for CD44^+^CD117^+^ cells with a cancer stem cell-like phenotype,^6^ we showed that intraperitoneal delivery of the CXCR4-A-armed vaccinia was more efficacious in inhibiting tumor growth compared with treatment with the soluble CXCR4-A (sCXCR4-A) counterpart or a systemic injection of the armed virus because of higher accumulation of the antagonist in tumors rather than in systemic tissues. The armed virotherapy treatment increased intratumoral accumulation of CD103^+^ DCs, and their subsequent expansion by injection of the FL cytokine enhanced infiltration of antigen-experienced CD8^+^ TILs and provided a platform for a booster immunization with the WT1 peptide-based vaccine delivered with polyriboinosinic:polyribocytidylic acid (poly(I:C)) as an adjuvant. Our studies revealed that expansion of intratumoral CD103^+^ DCs following CXCR4 antagonist-armed oncovirotherapy treatment represents a viable approach for *in situ* therapeutic vaccination to effectively bolster antitumor immune responses.

## Results

### Inhibition of ID8-T Ovarian Tumor Growth after Intraperitoneal or Systemic Injection of CXCR4-A Delivered as a Soluble Antagonist or by Oncolytic Virotherapy

**Figure 1:**
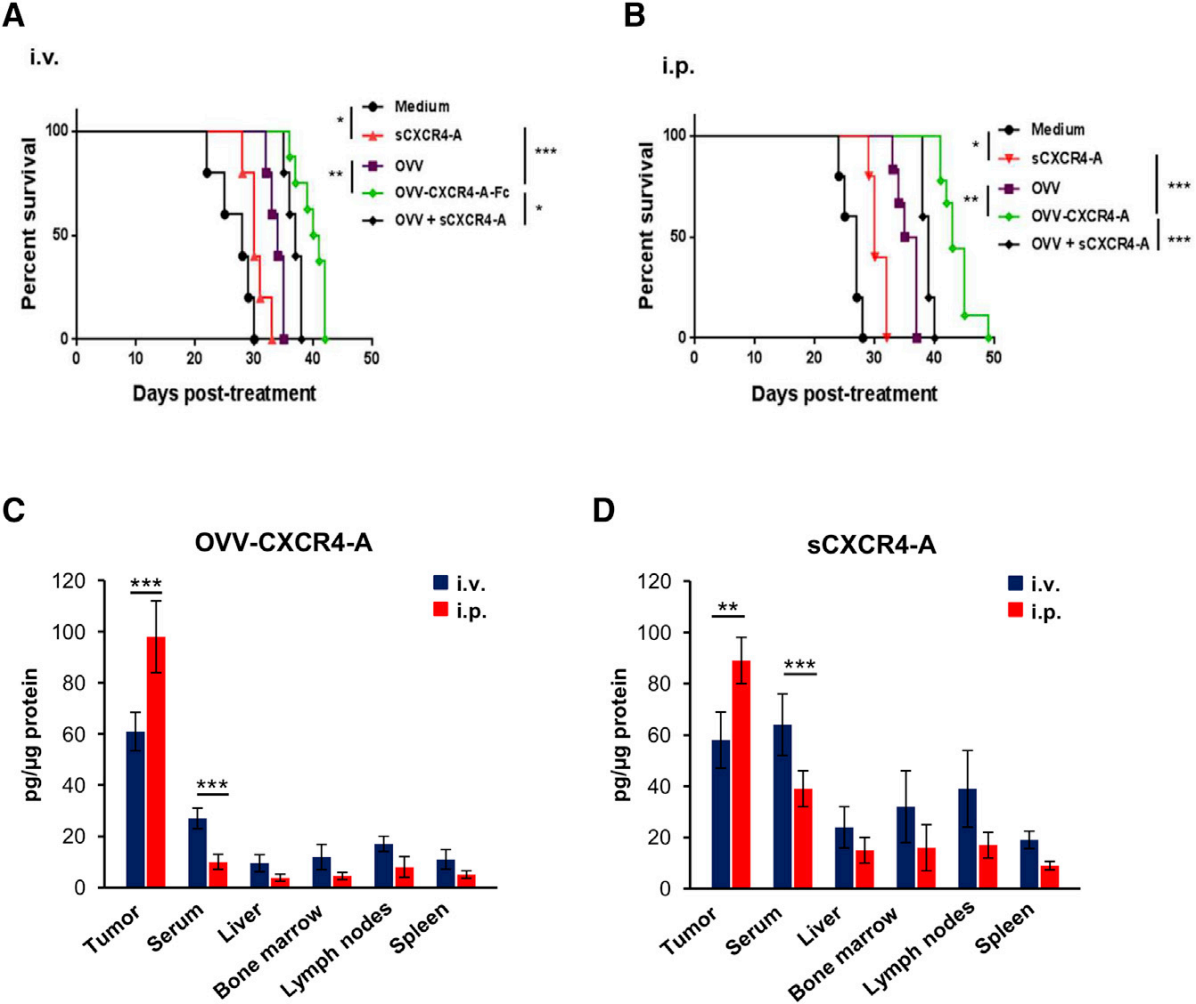
Inhibition of ID8-T Tumor Growth and Accumulation of sCXCR4-A in Peritoneal Washes of Tumor-Bearing Mice, Sera, and Lymphoid Organs after i.v. or i.p. Delivery of OVV-CXCR4-A and sCXCR4-A (A and B) C57BL/6 female mice (n = 5–10 mice/group) were challenged i.p. with 3 × 10^5^ ID8-T tumor cells and treated with sCXCR4-A (10 μg/injection for 7 days), OVV or OVV-CXCR4-A (10^8^ PFU), or OVV and sCXCR4 combinations injected i.v. (A) or i.p. (B) 10 days after tumor challenge. Control mice were treated with RPMI-1640 medium. Tumor progression was monitored by bioluminescence. Kaplan-Meier survival plots were prepared, and significance was determined using the log rank method. *p < 0.05, **p < 0.01, ***p < 0.001. (C and D) Accumulation of sCXCR4-A in peritoneal washes, sera, and lymphoid organs of tumor-bearing mice after i.v. or i.p. delivery of OVV-CXCR4-A (C) or sCXCR4-A (D) to ID8-T tumor-bearing mice. Concentrations of sCXCR4-A in sera, peritoneal washes (denoted as tumors), livers, BM, lymph nodes, and spleens were determined on day 8 after treatment by ELISA after normalization to total protein content. Data are presented as the mean ± SD of five mice per group. **p < 0.01, ***p < 0.001.

We first assessed the effect of intravenous (i.v.) or intraperitoneal (i.p.) delivery of soluble and virally delivered CXCR4-A, expressed in-frame with the murine Fc fragment of immunoglobulin G2a (IgG2a; OVV-CXCR4-A), in C57BL/6 mice challenged i.p. with a highly metastatic syngeneic ovarian cancer cell line (ID8-T). The treatment was initiated 10 days after tumor challenge and consisted of a single injection (10^8^ plaque-forming units (PFUs)/mouse) of OVV-CXCR4-A or control EGFP-expressing virus (OVV). To determine the contribution of the antagonist alone to controlling tumor growth, additional tumor-bearing mice were treated for 7 days with sCXCR4-A (10 μg/injection) delivered i.v. or i.p. or were injected with RPMI-1640 medium (control mice). Inhibition of tumor growth, quantified by bioluminescence imaging, revealed rapid tumor progression in untreated control mice (Figures S1A and S1B), with animals reaching a humane endpoint within 4 weeks of challenge (Figures 1A and 1B). Systemic delivery of OVV-CXCR4-A reduced tumor growth and extended survival compared with untreated controls (p < 0.001) or animals treated with the unarmed virus (p = 0.002; Figure S1A). On the other hand, systemic injection of sCXCR4-A demonstrated only modest effects in controlling tumor spread and extended survival by ∼1 week compared with control tumor-bearing mice. The antitumor effects of the virus or soluble antagonist were more pronounced after i.p. treatment (Figure S1B). I.p. delivered OVV-CXCR4-A controlled tumor growth for 4–5 weeks, and then the tumor progressed, extending survival by over 14 days compared with mice treated with sCXCR4-A (p < 0.001; Figure 1B) or by ∼10 days compared with the OVV-treated counterparts. A combination of the control virus and sCXCR4-A delivered either i.v. or i.p. was more efficacious in reducing tumor growth (Figures S1A and S1B) and increased survival compared with each treatment alone (p < 0.05; Figures 1A and 1B). The combination, however, did not achieve higher efficacy compared with a single treatment with OVV-CXCR4 (Figures 1A and 1B). This could be due to variations in the distribution of sCXCR4-A in the TME after injection compared with close contact of the antagonist with tumor stromata and cancer cells after being released from OVV-CXCR4-A-infected cancer cells. Differences in the level and physical contact of sCXCR4-A with cancer cells could directly affect tumor growth through induction of apoptosis after binding to CXCR4-expressing ID8-T cells, followed by phagocytosis of tumor cell debris by DCs (Figures S2A and S2B), a process required for induction of antitumor immune responses.^8^ Thus, the more efficacious inhibition of ID-8-T tumor growth by i.p. delivery of the antagonist, either by the virus or in a soluble form, could be associated with higher concentrations of sCXCR4-A in the tumor compared with systemic delivery, as measured on day 8 after treatment (p < 0.01; Figures 1C and 1D). The i.p. treatment also resulted in background levels of the antagonist in sera or other organs, which was in contrast to ∼2-fold higher levels of sCXCR4-A detected in sera and lymphoid organs of mice after systemic delivery. The higher concentrations of sCXCR4-A in the blood and systemic tissues after i.v. injection compared with i.p. delivery were associated with ∼10% increased numbers of leukocytes in the peripheral blood on days 8 and 15 before returning to baseline on day 30, although the treatment had no effect on red blood cell and platelet counts (Figures S3A–S3C).

### Reduction of Intratumoral Immune Suppression and Enhanced Infiltration of CD103^+^ DCs after OVV-CXCR4-A Treatment

**Figure 2:**
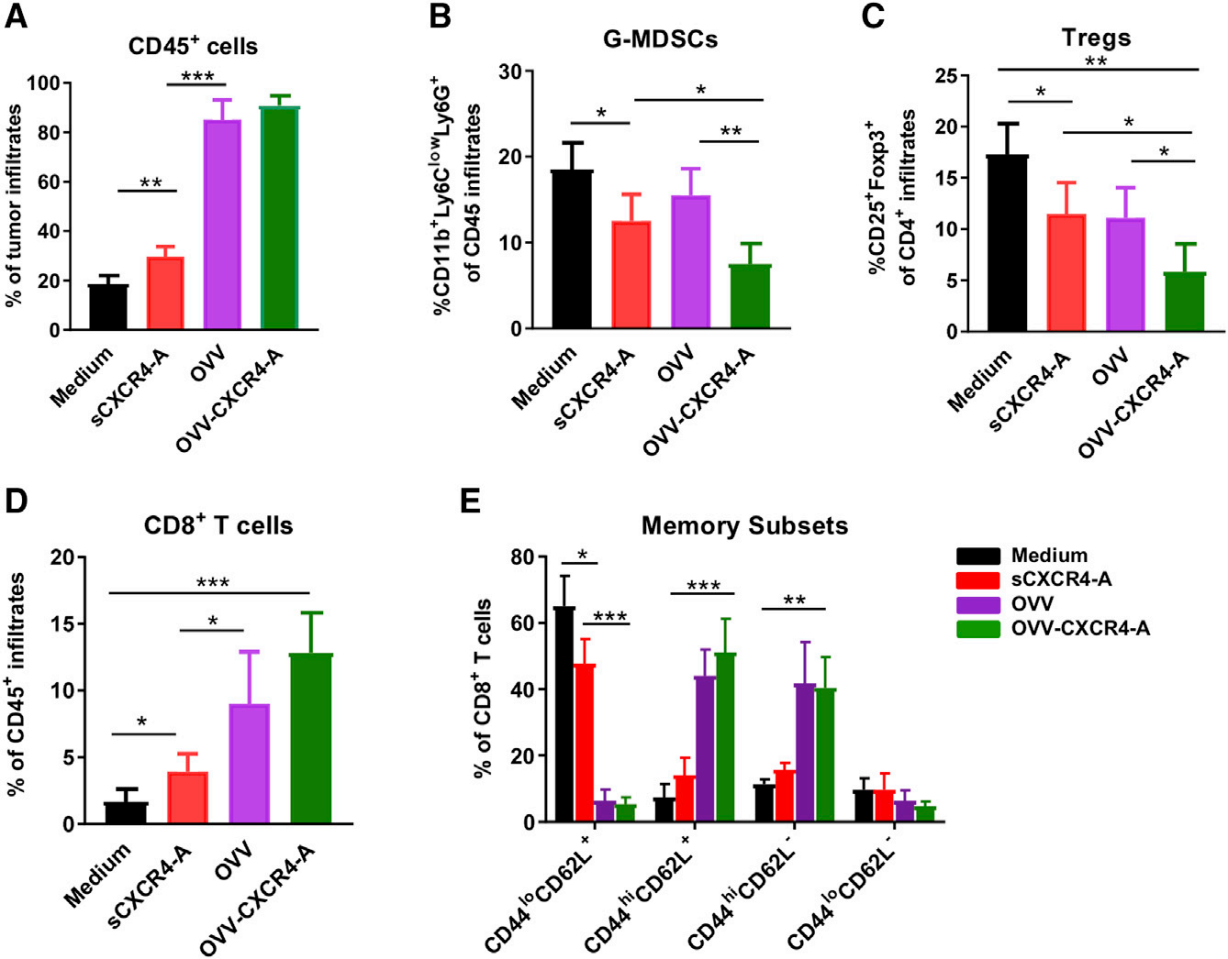
Increased Inflammation and Inhibition of the Immunosuppressive Network in the TME by Virally Delivered CXCR4-A (A–D) Accumulation of leukocytes (CD45^+^) (A), G-MDSCs (CD11b^+^Ly6G^hi^Ly6C^lo^) (B), Tregs (CD4^+^CD25^+^Foxp3^+^) (C), and CD8^+^ T cells (D) in peritoneal washes of ID8-T ovarian tumor-bearing mice was analyzed by flow cytometry 8 days after treatment. (E) Memory subsets of CD8^+^ T cells were analyzed with mAbs specific for CD44 and CD62L antigens. Background staining was assessed using isotype control antibodies. Data are mean ± SD of three or four independent experiments. *p < 0.05, **p < 0.01, ***p < 0.001.

Previous studies have shown that virally delivered CXCR4 antagonist blocks the CXCL12/CXCR4 axis involved in tumor progression by inhibiting local immunosuppression.^6^, ^7^, ^8^, ^20^ Therefore, we next investigated the effects of sCXCR4-A and OVV-CXCR4-A treatments on intratumoral accumulation of granulocyte-like myeloid-derived suppressor cells (G-MDSCs) and Tregs within the TME by flow cytometry analyses performed 8 days later, which roughly corresponded to the termination of viral replication *in vivo*.^6^ As shown in Figure 2A, the frequencies of tumor-infiltrating CD45^+^ leukocytes in tumor-bearing mice after virotherapy treatments were ∼4-fold higher compared with those in the untreated or sCXCR4-A-treated counterparts. The antagonist, delivered i.p. as a soluble protein or secreted from virally infected tumor cells, reduced the accumulation of immunosuppressive CD11b^+^Ly6C^low^Ly6G^high^ G-MDSCs compared with the untreated and OVV-treated counterparts (Figure 2B; p = 0.03 and p = 0.006, respectively), and also inhibited accumulation of CD4^+^CD25^+^Foxp3^+^ Tregs (Figure 2C; p < 0.05). Inhibition of the immunosuppressive network within the TME contributed to increased accumulation of CD8^+^ TILs, which were detected after sCXCR4-A delivery (p = 0.02) and increased by over 3-fold after OVV or OVV-CXCR4-A treatment (Figure 2D; p < 0.01). The virotherapy-expanded CD8^+^ TILs consisted mostly of antigen-experienced (CD44^hi^CD62L^+^ and CD44^hi^CD62L^−^) cells with less than 5% naive (CD44^lo^CD62L^+^) and double-negative cells (Figure 2E), which was in contrast to the predominantly naive phenotype of CD8^+^ TILs in untreated mice. Treatment with sCXCR4-A increased the frequencies of CD44^hi^CD62L^+^ and CD44^hi^CD62L^−^ CD8^+^ cells compared with control mice, but the changes were not significant.

**Figure 3:**
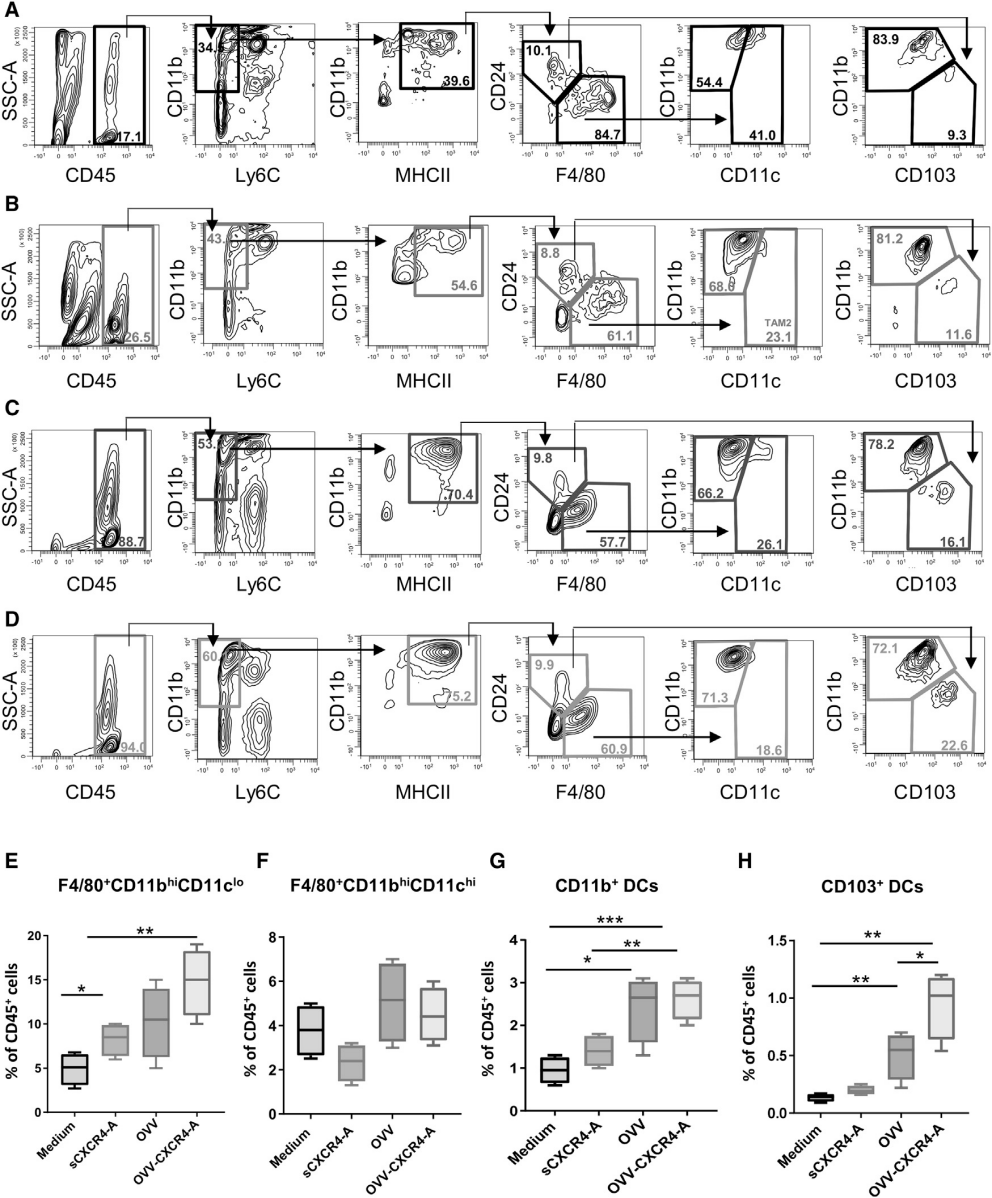
Intratumoral Infiltration of CD103^+^ DCs after i.p. Treatment with Soluble or Virally Delivered CXCR4-A (A–D) Representative flow cytometry staining and gating of myeloid cell populations infiltrating the peritoneal cavities of ID8-T-challenged mice treated with medium (A), sCXCR4-A protein (B), OVV (C), and OVV-CXCR4-A (D). (E–H) Relative proportions of tumor-infiltrating F4/80^+^CD11b^hi^CD11c^lo^ (E), F4/80^+^CD11b^hi^CD11c^hi^ (F), CD11b^+^ DCs (G), and CD103^+^ DCs (H) are depicted as percentages of total CD45^+^ cells. Results are presented as mean ± SD from three or four independent experiments. *p < 0.05, **p < 0.01, ***p < 0.001.

The increased percentages of CD8^+^ TILs after oncovirotherapy treatment were associated with higher infiltration of tumor-associated macrophages (TAMs) and DCs, profiled within the CD45^+^ compartment using multi-color flow cytometry and a progressive gating strategy.^33^ As shown in Figures 3A–3D, subgating all CD45^+^ hematopoietic cells by the myeloid-specific marker CD11b that were Ly6C-negative allowed removal of neutrophils (CD11b^+^Ly6C^lo^) and monocytes (CD11b^+^Ly6C^hi^). Within the CD11b^+^MHCII^+^ subset, macrophages were distinguished from DCs based on CD24^lo^ and F4/80^hi^ expression, and because neither marking alone is sufficient to make this distinction,^33^ these two populations were analyzed separately. Staining of the F4/80^hi^CD24^lo^ cells with CD11b and CD11c showed that the majority of macrophages exhibited the CD11b^hi^CD11c^lo^ phenotype, captured by the TAM1 subset of macrophages,^33^ with only small proportions being double-positive for both antigens and CD11c^hi^CD11b^hi^ in all treatment groups. The results, presented as the percentages of TAMs within CD45^+^ cells, revealed that the relative proportions of F4/80^+^CD11b^hi^CD11c^lo^ cells were higher in treatment groups compared with control mice (Figure 3E; p < 0.05), whereas no significant differences were observed in the proportions of F4/80^+^CD11c^hi^CD11b^hi^ cells (Figure 3F). This was in contrast to increased percentages of CD11b^+^ and CD103^+^ DCs within the F4/80^lo^CD24^hi^ population after virotherapy treatments (Figures 3G and 3H; p < 0.05) with significantly higher numbers of CD103^+^ DCs in OVV-CXCR4-A-treated tumors compared with OVV-treated counterparts (p = 0.04).

### FL-Mediated Expansion of Intratumoral CD103^+^ DCs Inhibited Tumor Growth and Augmented Infiltration of CD8^+^Ly6C^+^ TILs

**Figure 4:**
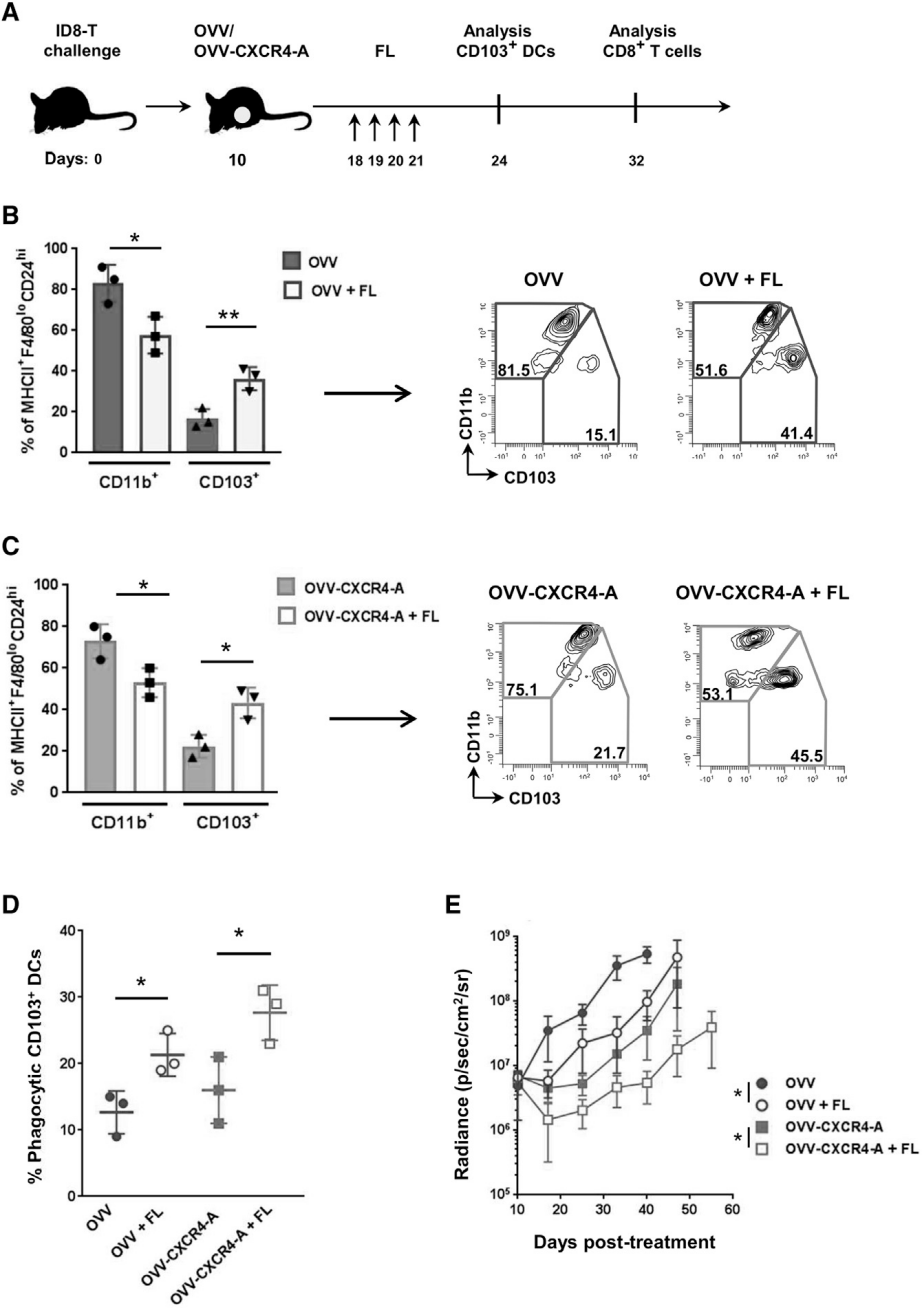
Expansion of Intratumoral CD103^+^ DCs by Local Delivery of the FL Cytokine Enhances the Efficacy of Oncolytic Virotherapy Treatment (A) Graphical timeline of the treatment scheme in ID8-T tumor-bearing mice. C57BL/6 mice were injected i.p. with 3 × 10^5^ ID8-T cells. Treatment with OVV or OVV-CXCR4-A (10^8^ PFU delivered i.p.) was initiated 10 days later. To expand CD103^+^ DCs, FL was injected i.p. at 5 μg/injection for 4 consecutive days, beginning on day 8 after virotherapy treatment. Percentages of CD11b^+^ and CD103^+^ DCs in peritoneal washes of OVV- or OVV-CXCR4-A-treated, ID8-T-bearing mice (n = 3–5 mice/group) after i.p. delivered FL were analyzed 2 days later, whereas percentages of CD8^+^ TILs were assessed on day 32 by flow cytometry. (B and C) Relative proportions (left panel) and representative flow cytometry plots (right panel) of intratumoral CD11b^+^ and CD103^+^ DCs within MHCII^+^F4/80^lo^CD24^hi^ populations of myeloid cells infiltrating the peritoneal cavities of ID8-T tumor-bearing mice after OVV and FL treatment (B) as well as OVV-CXCR4-A and FL treatment (C). Results are presented as mean ± SD of four experiments. *p < 0.05, **p < 0.01. (D) FL-mobilized CD103^+^ DCs exhibited increased phagocytosis of tumor cell debris. CD45^+^ leukocytes isolated from peritoneal cavities of ID8-T tumor-bearing mice 2 days after treatment with OVV or OVV-CXCR4-A alone or in combination with FL were cultured with OVV-treated and CellTracker-labeled ID8-T cancer cells. After overnight incubation, the capture of tumor-associated fluorescent debris by CD103^+^ DCs was analyzed by flow cytometry. Percentages of phagocytosis of virally treated tumor cell debris by CD103^+^ DCs are presented as mean ± SD of 3 experiments. *p < 0.05. (E) Progression of ID8-T tumor growth in mice (n = 5 mice/group) treated with OVV or OVV-CXCR4-A delivered alone or in combination with the FL cytokine was monitored by bioluminescence. Data points represent mean ± SD. *p < 0.05.

Given the profound association of intratumoral stimulatory DCs with patient outcome,^34^ we sought to determine whether expansion of CD103^+^ DCs in the TME would enhance the therapeutic efficacy of the combined treatment by promoting tumor antigen presentation and priming of T cells following virotherapy-mediated ICD.^35^ The formative cytokine for cDC1s, which include tumoral CD103^+^ DCs, is FL, which is predominantly produced by lymphocytes, notably natural killer cells in mouse and human tumors.^2^ Because the antitumor effect of oncolytic virotherapy is short-lasting because the virus is eliminated by the innate and adaptive immune responses, we hypothesized that the paucity of CD103^+^ DCs at the tumor site restricted the expansion of tumor-specific CD8^+^ T cells and, therefore, limited the efficacy of the viroimmunotherapy treatment. We therefore sought to determine whether expansion of intratumoral CD103^+^ DCs by local delivery of the FL growth factor^36^ would enhance the therapeutic efficacy of the combined treatment by promoting tumor antigen presentation and priming T cells following virotherapy-mediated ICD.^35^ As depicted in Figure 4A, 8 days after virotherapy treatment, tumor-bearing mice were injected i.p. with FL (5 μg/injection) for 4 days, and changes in tumor-infiltrating DCs were analyzed 2 days later by flow cytometry. As shown in Figures 4B and 4C, injection of the FL cytokine expanded over 2-fold (p < 0.05) the frequency of CD103^+^ DCs among the MHCII^+^F4/80^lo^CD24^hi^ cell population in both OVV and OVV-CXCR4-A-treated tumor-bearing mice (Figures 4B–4E). The combination treatment-expanded CD103^+^ DCs were able to engulf cellular debris from OVV-exposed ID8-T cells at higher levels compared with their virotherapy-expanded counterparts (Figure 4D; p ≤ 0.04), which is stringently required for mounting an immune response against dying tumor cells.^8^ The FL-mediated increases in CD103^+^ DCs in tumor-bearing mice were associated with decreased tumor growth (Figure 4E) compared with animals receiving monotherapy with OVV (p = 0.04) or OVV-CXCR4-A (p = 0.03).

**Figure 5:**
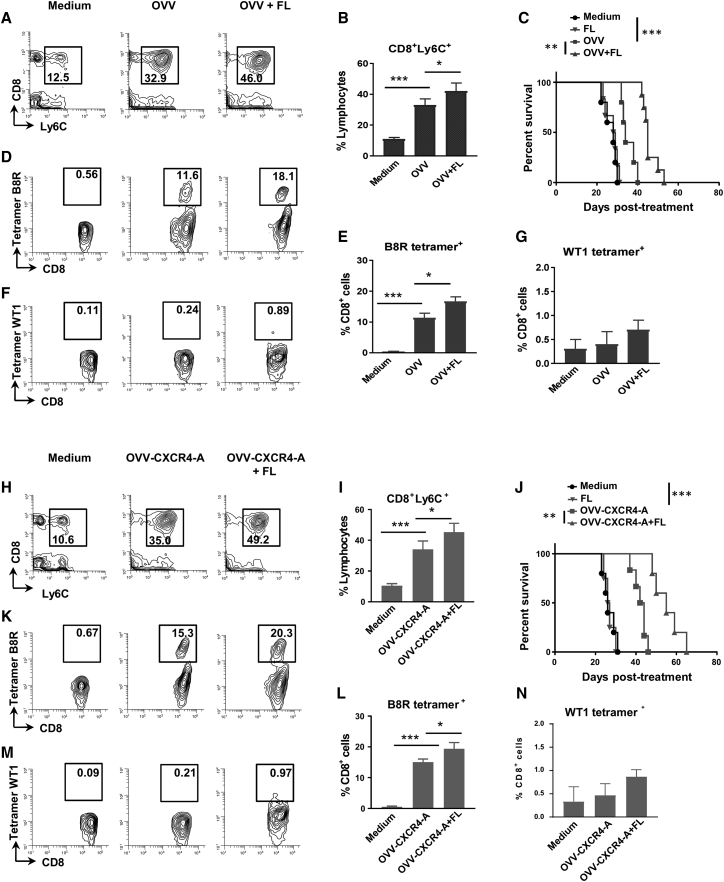
FL-Mediated Expansion of Intratumoral CD103^+^ DCs Inhibits Tumor Growth and Augments Infiltration of CD8^+^ TILs to Peritoneal Cavities of ID8-T Tumor-Bearing Mice (A and B) Evaluation of tumor-infiltrating CD8^+^Ly6C^+^ T cells in peritoneal washes after OVV treatment alone or in combination with the FL cytokine (n = 5 mice/group). Representative flow cytometry staining (A) and relative proportions of CD8^+^Ly6C^+^ TILs (B) are shown. (C) Survival of ID8-T tumor-bearing C57BL/6 mice (n = 5–10 mice/group) after OVV and OVV plus FL treatment combinations. Survival was defined as the point where mice were killed because of extensive tumor burden. Kaplan-Meier survival plots were prepared, and significance was determined using the log rank method. **p < 0.01, ***p < 0.001. (D and E) Representative flow cytometry staining (D) and relative proportions of a tumor-infiltrating B8R vaccinia virus-specific tetramer^+^ subset of CD8^+^ TILs (E). (F and G) Representative flow cytometry staining (F) and relative proportions of WT1 tetramer^+^CD8^+^ TILs (G). (H and I) Evaluation of tumor-infiltrating CD8^+^Ly6C^+^ T cells in peritoneal washes after OVV-CXCR4-A treatment alone or in combination with the FL cytokine (n = 3–5 mice/group). Representative flow cytometry staining (H) and relative proportions of the CD8^+^Ly6C^+^ TILs (I) regimen are shown. (J) Survival of ID8-T tumor-bearing C57BL/6 mice (n = 5–10 mice/group) after OVV-CXCR4-A and OVV-CXCR4-A plus FL treatment combination. Kaplan-Meier survival plots were prepared, and significance was determined using the log rank method. **p < 0.01, ***p < 0.001. (K and L) Representative flow cytometry staining (K) and relative proportions of a tumor-infiltrating B8R vaccinia virus-specific tetramer^+^ subset (L) after combined treatment with OVV-CXCR4-A and FL. (M and N) Representative flow cytometry staining (M) and relative proportions of WT1 tetramer^+^CD8^+^ TILs (N).

Because intratumoral infiltration of CD103^+^ DCs is one of the major requirements for establishing a T cell-inflamed tumor phenotype because of production of CXCL9 and CXCL10 chemokines, which promote recruitment of effector CXCR3^+^ CD8^+^ T cells,^37^ we next examined whether this mechanism could also be used to increase survival and bolster tumor-specific T cell responses following virotherapy. For the analysis, CD8^+^ T cells in the peritoneal cavities of control and virotherapy-treated mice were stained with antibodies specific to Ly6C antigen expressed on antigen-experienced T cells^38^ as well as tetramers specific for the vaccinia virus B8R protein (B8R-K^b^/TSYKFESV) and WT1 tumor antigen (WT1-2D^b^/RMFPNAPYL). Figures 5A and 5B show a more than 3-fold expansion of intratumoral CD8^+^Ly6C^+^ cells after OVV treatment compared with control mice (p < 0.001), and the numbers increased by ∼30% after FL delivery (p < 0.05). The increased percentages of antigen-experienced CD8^+^Ly6C^+^ TILs after OVV and FL combination treatment extended the median survival rate (45 days; Figure 5C) compared with OVV-treated and control groups of mice (33 and 27 days, respectively). Because injection of the FL cytokine into untreated mice did not affect the survival rate, it appears that virotherapy-mediated accumulation of intratumoral DCs and changes in the TME are required for the FL-mediated antitumor effect. Over 10% of CD8^+^ TILs in mice that received oncolytic virotherapy treatments were positive for the B8R-K^b^/TSYKFESV vaccinia-specific tetramer, with additional increases in the percentages of tetramer-positive cells measured after FL delivery (Figures 5D and 5E). However, despite significant increases in the frequencies of CD8^+^Ly6C^+^ TILs, including those that were directed against the viral antigen, percentages of WT1 tetramer^+^CD8^+^ T cells were at background levels after oncolytic virotherapy and FL combination treatment (Figures 5F and 5G). CD8^+^Ly6C^+^ T cell responses were increased after the OVV-CXCR4-A and FL treatment combination compared with tumor-bearing mice treated with the control virus and FL (p = 0.016; Figures 5H and 5I) and were associated with an increased survival rate (Figure 5J). The higher percentages of B8R tetramer^+^CD8^+^ TILs in OVV-CXCR4-A-treated mice compared with those receiving the control virus with or without FL treatment (p < 00.4; Figure 5K) also indicated that the release of CXCR4-A from virally infected tumor cells did not interfere with migration of antigen-specific T cells to the TME, consistent with minimal expression of CXCR4-A on differentiated effector and effector memory T cells.^39^ It is also notable that the percentages of B8R tetramer^+^CD8^+^ T cells in spleens and tumors, measured after single or multiple (3 times) deliveries of the oncolytic viruses, were similar (Figures S4A–S4C), possibly because of acquired resistance of residual tumors to repeated viral infections. However, despite the higher frequencies of B8R-K^b^/TSYKFESV tetramer^+^CD8^+^ TILs after OVV-CXCR4-A and FL treatment compared with those generated using the control virus combination (p < 0.05), the percentages of WT1 tetramer^+^CD8^+^ T cells still remained at background levels (Figure 5L). Therefore, we hypothesized that a weak expression level of the WT1 protein in ID8-T cancer cells, together with an excess of highly phagocytic macrophages in the TME, which compete for antigen availability at the tumor site, could limit the ability of CD103^+^ DCs to prime and activate sufficient numbers of WT1 tetramer^+^CD8^+^ T cells. We next investigated whether boosting the load of WT1 antigen at the tumor site with an adjuvanted WT1 peptide-based vaccine would enhance the frequencies of WT1 tetramer^+^CD8^+^ TILs.

### Generation of WT1-Specific CD8^+^ TILs by an Adjuvanted WT1-Peptide Vaccine Delivered after Oncovirotherapy and FL Treatment Required Batf3-Driven CD103^+^ DCs

**Figure 6:**
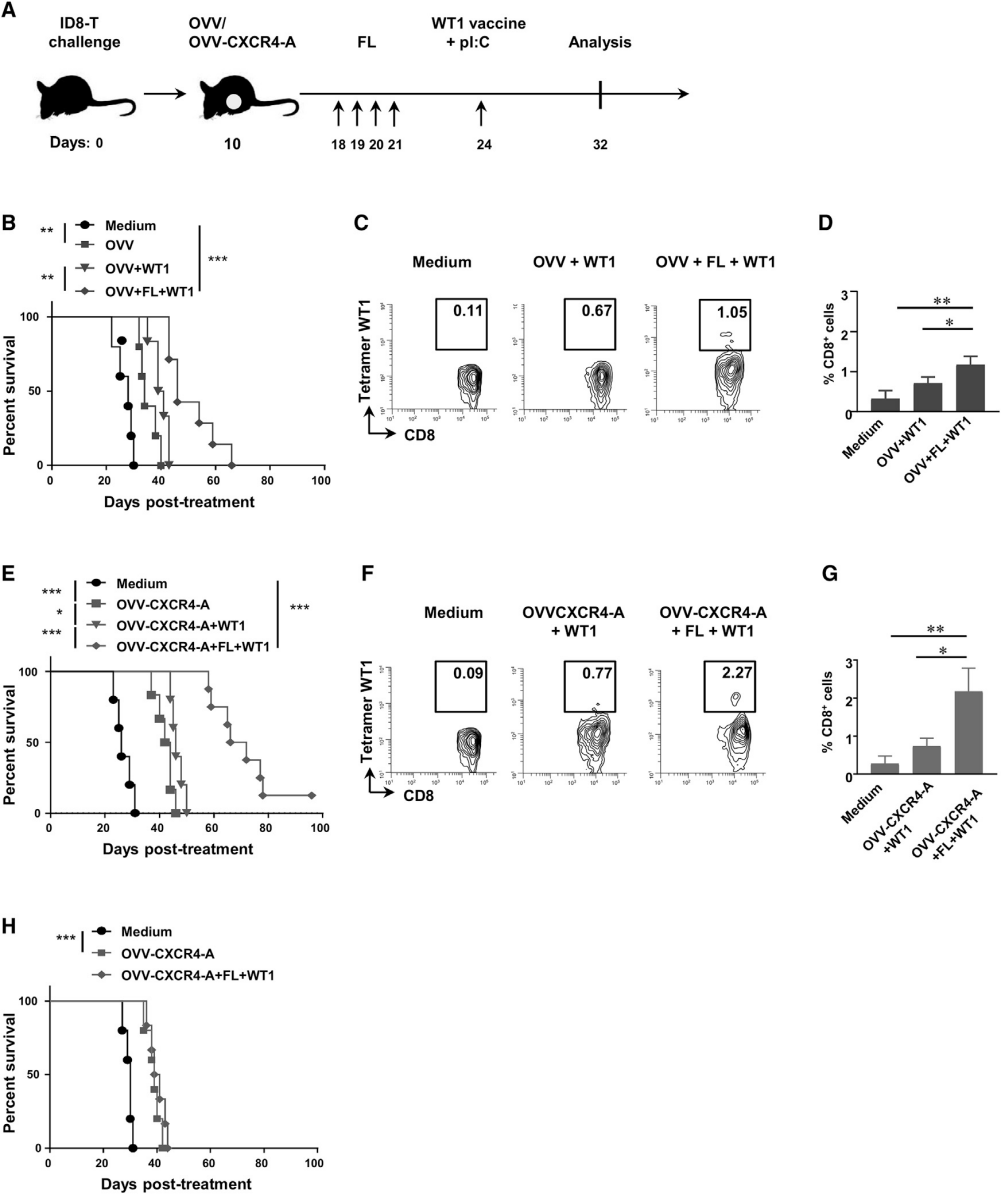
An Adjuvanted WT1 Vaccine Delivered after Oncovirotherapy and FL Treatment Combination Generates WT1-Specific CD8^+^ TILs and Requires Batf3-Driven CD103^+^ DCs (A) Graphical timeline of the treatment scheme in ID8-T tumor-bearing mice. C57BL/6 mice were injected i.p. with 3 × 10^5^ ID8-T cells. Treatment with OVV or OVV-CXCR4-A (10^8^ PFU delivered i.p.) was initiated 10 days later. To expand CD103^+^ DCs, FL was injected i.p. at 5 μg/injection for 4 consecutive days, beginning on day 8 after virotherapy treatment. The WT1-specific peptide was delivered i.p. (50 μg/injection) with poly(I:C) (p(I:C); 50 μg/injection) on day 3 after the last FL delivery. (B) Survival of ID8-T tumor-bearing mice (n = 5–10 mice/group) after WT1 immunization of OVV- and OVV plus FL-treated mice. Kaplan-Meier survival plots were prepared, and significance was determined using the log rank method. **p < 0.01. (C and D) Representative flow cytometry staining (C) and relative proportions of WT1 tetramer^+^CD8^+^ TILs (D) after combined treatment of OVV and WT1 vaccine as well as OVV and FL treatment followed by WT1 vaccination (n = 4–5 mice/group). (E) Survival of ID8-T tumor-bearing mice (n = 5–10 mice/group) after WT1 immunization of OVV-CXCR4-A and OVV-CXCR4-A plus FL-treated mice. *p < 0.05, ***p < 0.001. (F and G) Representative flow cytometry staining (F) and relative proportions of WT1 tetramer^+^CD8^+^ TILs (G) after combined treatment of OVV-CXCR4-A and WT1 vaccine and OVV-CXCR4-A plus FL treatment followed by WT1 vaccination (n = 4–5 mice/group). (H) Survival of ID8-T tumor-bearing *Batf3*^*−/−*^ female mice (n = 5) after treatment with OVV-CXCR4-A alone or in combination with FL and the WT1 adjuvanted vaccine. Kaplan-Meier survival plots were prepared, and significance was determined using the log rank method. ***p < 0.001.

The WT1-specific peptide containing H2-IA^b^-restricted CRYGPFGPPPSQAS and H2-D^b^-restricted RMFPNAPYL epitopes^6^, ^8^ was injected i.p. to ID8-T-bearing mice (50 μg/injection) 3 days after FL delivery (Figure 6A) in combination with poly(I:C) (50 μg/injection), which binds to TLR3 expressed on CD103^+^ DCs^40^ and induces type I IFN production and DC maturation.^41^, ^42^ Additional groups of tumor-bearing mice received only virotherapy treatments before immunization to determine the importance of FL-expanded CD103^+^ DCs in the induction of tumor-antigen-specific T cells and inhibition of tumor growth. As shown in Figure 6B, vaccination of mice after OVV and FL treatment combination exhibited potent antitumor activities, extending survival by about 15 days compared with mice treated with the virus (p < 0.001) and by 7–8 days compared with the virus and WT1 vaccine (p = 0.006). This regimen also elicited measurable WT1-specific CD8^+^ T cell responses compared with those induced by vaccination without prior FL treatment (p = 0.03; Figures 6C and 6D). CXCR4-A-armed virotherapy followed by FL-mediated expansion of CD103^+^ DCs prior to vaccination was most effective in inhibiting tumor growth (median survival of 69 days; Figure 6E) and inducing WT1-2D^b^/RMFPNAPYL tetramer^+^CD8^+^ TILs (Figures 6F and 6G). Additional experiments performed in *Batf3*^*−/−*^ knockout mice deficient for both CD103^+^ and CD8α^+^ DCs^29^, ^43^ revealed an absence of the WT1 vaccine-mediated protective responses (Figure 6H), stressing the need for CD103^+^ DCs at the tumor site for induction of antitumor protective immune responses.

## Discussion

As cancer therapies continue to evolve and incorporate immunotherapy as an integral aspect of treatment, developing approaches that potentiate the induction of ICD and overcome non-T cell inflamed tumors will be important to realizing increased treatment efficacy. Here we showed that locoregional delivery of the CXCR4-A-armed virus is more efficacious in inhibiting orthotopic growth of ovarian tumors than i.v. injection of the unarmed counterpart, possibly because of a higher accumulation of the antagonist in the tumor than in systemic tissues. It also appears that distribution of the CXCR4 antagonist in the TME and its vicinity to both stromal and cancer cells play an important role in blocking the CXCL12/CXCR4 signaling pathway. For example, physical contact of the antagonist with the target can be more efficacious when it is released from virally infected cancer cells directly to the TME than delivered by injection because the latter form of delivery may not facilitate effective penetration in the tumor tissue. This hypothesis is consistent with higher inhibition of tumor growth by i.p. delivery of OVV-CXCR4-A virus than by injection of the soluble antagonist with a control virus by the same route. The results of our studies are in agreement with the recent work by Chen et al.,^20^ demonstrating that high concentrations of localized CXCR4-A in the TME decreases immunosuppression associated with enhanced infiltration of CD8^+^ TILs and inhibition of tumor growth. This, together with the findings that the TME may regulate clonal expansion of cancer-specific T cells^44^ and that CD8^+^ T cell proliferative responses are orchestrated by CD103^+^ Baft3-dependent DCs^32^ suggest dependence of T cell-mediated tumor regression on the intratumoral presence of CD103^+^ DCs. Thus, therapeutic interventions that enhance infiltration of intratumoral stimulatory DCs and their capacity for driving T cell proliferation may contribute to tumor control. Among such strategies are interventions that target intratumoral TAMs and MDSCs and lead to reduced tumor burdens in preclinical models in both T cell-dependent and T cell-independent ways. For instance, inhibiting chemokine receptor type 2 (CCR2),^45^ colony-stimulating factor-1 receptor (CSF-1R),^45^, ^46^ and GM-CSF^47^ in preclinical models of melanoma and pancreatic, breast, and prostatic carcinoma increased intratumoral T cells and controlled tumor growth, especially when combined with anti-CTLA-4 or anti-PD-1/PD-L1. Although these studies did not determine whether the increases in T cells were a consequence of enhanced viability or proliferation, they emphasize that elements of the TME regulate the accumulation of effector T cells. In addition, the distribution of intratumoral CXCL12, which correlates inversely with that of T cells, suggests that CXCL12 is involved in T cell exclusion based on the antitumor outcome of inhibiting CXCR4. The results of our recent studies further emphasize this assumption and demonstrate that CXCR4-A-armed oncolytic virotherapy treatment was associated with increases in intratumoral accumulation of CD103^+^ DCs and that its efficacy could be further boosted by FL-mediated expansion of CD103^+^ DCs.

By inducing ICD and antigen release at the tumor site via viral oncolysis with simultaneous reprogramming of the TME, the armed virotherapy is personalized and can be combined with tumor-specific vaccines^48^, ^49^ after increasing the intratumoral infiltration of CD103^+^ DCs by injection of the FL cytokine. As demonstrated here, intratumoral accumulation of CD103^+^ DC populations at the tumor site served as a platform for the adjuvanted WT1-specific peptide vaccine booster, leading to generation of WT1 tetramer^+^CD8^+^ TILs and increases in overall survival. This approach could be used with a variety of tumor-associated antigens as an “off-the-shelf” product for immunization or with personalized neoantigen-specific epitopes, the presence of which has been shown to correlate with expression of immune-related genes and efficacy of checkpoint inhibitor therapy.^50^ Thus, the described “*in situ* vaccination” strategy is feasible and effective in inducing and amplifying T cell responses to tumor antigens. Because a high mutational burden has been associated with an increased neoantigen load and TILs, which improved clinical outcomes and survival seen in patients with tumors, incorporating novel peptide sequences that result from protein-changing somatic mutation in cancer cells will be of utmost value.^51^ The ability of intratumoral virotherapy to broaden the neoepitope spectrum when delivered with systemic PD-1 checkpoint inhibition, resulting in improved antitumor efficacy,^52^ is consistent with the observation that oncolytic viruses may not only be used as direct tumor therapy but may also serve as a method to validate the responsiveness of T cells to predicted neoepitopes.^53^

The CXCR4-A oncolytic virotherapy-generated immunogenic tumor cell “cargo” for DC loading has the potential to be further enhanced by combination with ICD-inducing chemotherapeutic agents, such as doxorubicin, to promote improved antigen presentation to T cells^54^ because of a synergistic interaction between OVV and doxorubicin.^8^ This synergy could increase the amount of tumor antigens for cross-priming and broaden the diversity of danger-associated molecular patterns (DAMPs). We also found that CXCR4-A, by binding to its cognate receptor on cancer cells and inducing apoptosis, was capable of increasing phagocytosis of tumor cell debris by DCs and, therefore, appears to indirectly improve the efficacy of virotherapy. This effect could be further augmented through an interaction with the Fcγ receptors (FcγRs) on phagocytes because the antagonist, expressed as a fusion protein with the Fc portion of IgG2a, has been shown to eliminate tumor cells through the antibody-dependent cellular cytotoxicity (ADCC) mechanism,^6^, ^7^ helping to achieve the desirable induction of antitumor immunity. In such a context, high concentrations of sCXCR4-A after locoregional delivery could be relevant in immunotherapies of cancer cells with deregulated type I IFN signaling pathways^55^ because FcγR-mediated antibody-dependent cellular phagocytosis bypasses the need for canonical phagocytic determinants. Such IgG-bound target cells can be efficiently processed, and the resulting tumor antigens can be used for cross-presentation by antigen-presenting cells (APCs), enhancing the repertoire of cancer antigen-directed T cell responses.^56^

Because the absence of CD103^+^ DCs in the TME may be a critical rate-limiting step for initiating endogenous CD8^+^ T cell responses against cancer,^31^ our results argue that CXCR4-A-armed virotherapy followed by FL treatment is effective in the expansion of intratumoral CD103^+^ DCs. The observed lack of interference of the CXCR4-A with DC infiltration is in agreement with previous studies, which showed that trafficking of DCs occurs in a coordinated, stepwise fashion, with CXCR4 and CXCL12 promoting the retention of pre-DCs in the BM but not migration to peripheral tissues and regional lymph nodes, which are directed by CCR2/CX3CR1 and CCR7, respectively.^57^ Similarly, the lack or minimal expression of CXCR4 on differentiated effector T cells^58^ explains the relatively high numbers of CD8^+^ TILs expressing the Ly6C antigen, known to be associated with the effector and effector memory phenotypes.^38^ Furthermore, the background levels of the CXCR4 antagonist in the blood and systemic tissues after i.p. treatment with OVV-CXCR4-A precluded any meaningful interference with the CXCR4-CCR5 interaction at the immunological synapse during T cell activation by APCs,^59^ despite high CXCR4 expression on naive and central memory T cells (T_CM_).^58^ It should also be realized that CXCR4-A-armed virotherapy treatment may have a profound effect on the induction of immune cells exhaustion, in view of recent studies showing that CXCR4 inhibition improves responses to immune checkpoint blockers in mice bearing metastatic breast cancers^20^ as well as decreases CD4^+^ T cell exhaustion and improves survival in a murine model of polymicrobial sepsis.^39^ Altogether, our study identifies combination therapies to potentiate ICD as well as the recruitment of CD103^+^ DCs to tumor sites for an effective *in situ* vaccination, which holds promise for the development of more efficacious treatments for cancer patients.

## Materials and Methods

### Animals and Cell Lines

Female C57BL/6 mice were obtained from Charles River Laboratories (Wilmington, MA, USA). B6.129S(C)-*Batf3*^*tm1Kmm*^/J mice were purchased from The Jackson Laboratory (Sacramento, CA, USA). Experimental procedures were performed in compliance with protocols approved by the Institutional Animal Care and Use Committee of the Roswell Park Comprehensive Cancer Center (RPCCC, Buffalo, NY, USA). The parental ID8 mouse ovarian epithelial cell line, derived from spontaneous malignant transformation of C57BL/6 MOSE cells,^60^ and its metastatic variant ID8-T were established in our laboratory at the RPCCC.^6^ Human HuTK^−^ 143 fibroblasts, human cervical carcinoma HeLa cells, and the African green monkey cell line CV-1 were obtained from the American Type Culture Collection (Manassas, VA, USA).

### Viruses

All vaccinia viruses used in this study were of the Western Reserve strain, with disrupted thymidine kinase and vaccinia growth factor genes for enhanced cancer cell specificity. The generation and characterization of OVVs expressing EGFP, the Fc portion of murine IgG2a, and CXCR4-A in the context of the Fc portion of murine IgG2a have been described.^7^ The CXCR4-A fusion protein was collected in supernatants of infected HuTK^−^ 143 cells and purified on a protein G column (GE Healthcare Life Sciences, Pittsburgh, PA, USA) as described.^7^

### ELISA

Concentrations of the soluble CXCR4-A protein in sera, cell lysates from systemic tissues, and peritoneal washes of tumor-bearing mice were measured by ELISA on day 8 after treatment using plates coated with a recombinant human CXCR4 protein, MEGISIYTSDNY TEEMGSGDYDSMKEPCFREENANFNKIFLPTIYS (Abcam, Cambridge, MA, USA), followed by incubation with goat anti-mouse Fc portion-specific horseradish peroxidase (HRP)-conjugated antibody (Sigma-Aldrich, St. Louis, MO, USA), and the reaction was developed with 1-Step Ultra TMB-ELISA reagent (Thermo Fisher Scientific, Grand Island, NY, USA). In parallel, protein levels in each sample were determined by the Bradford method with protein assay dye reagent (Bio-Rad, Hercules, CA, USA).

### *In Vitro* Phagocytosis Assays

CD45^+^ leukocytes isolated from peritoneal cavities of ID8-T-bearing mice 2 days after virotherapy treatment alone or in combination with FL were analyzed for their ability to engulf tumor cell debris from virally treated cultures. ID8-T cells were labeled with CellTracker Blue CMF_2_HC and treated with OVV at an MOI of 1 for 24 h before incubation with DCs (1:1 ratio) for 12 h. Tumor cells were treated with UV light (365 nm for 3 min) in the presence of 10 μg/mL psoralen to inactivate the virus. After overnight incubation, the capture of tumor-associated fluorescent debris by CD103^+^ DCs was analyzed by flow cytometry. For some experiments, BM cells were flushed from the tibiae and femora of C57BL/6 mice and cultured in medium supplemented with 10 ng/mL of GM-CSF for 6 days as described.^61^ After 7 days, non-adherent and loosely adherent cells were harvested, washed, and co-cultured with tumor cells labeled with CellTracker Blue CMF_2_HC (Thermo Fisher Scientific, 1:1 ratio) for 12 h.

### Treatments of Established Tumors

C57BL/6 mice (n = 5–10) were injected i.p. with 3 × 10^5^ ID8-T cells. Treatments with sCXCR4-A (10 μg/injection for 7 days), OVV, and OVV-CXCR4-A (10^8^ PFU), delivered i.v. or i.p., were initiated 10 days later. Tumor progression was monitored by bioluminescence imaging using the Xenogen IVIS Imaging System (PerkinElmer, Waltham, MA, USA) as described.^8^ Control mice received RPMI-1640 medium or UV-inactivated virus. At the end of the experimental period, corresponding to the development of bloody ascites in control mice, tumor-bearing mice were sacrificed, and organs were examined for tumor development and metastatic spread. For *in situ* immunization of ID8-T tumor-bearing mice, the FL cytokine (BioLegend, San Diego, CA, USA) was delivered i.p. (5 μg/injection) for 4 consecutive days, beginning on day 8 after virotherapy treatment, followed by the WT1-specific peptide vaccine (amino acids [aa] 175–202; CRYGPFGPPSQASSGOARMFPNAPYL; 50 μg/injection; GenScript, Piscataway, NJ, USA) and 50 μg/mouse of poly(I:C) (Sigma-Aldrich), delivered i.p. on day 3 after the last FL injection. Progression of tumor growth was analyzed by bioluminescence.

### Flow Cytometry

The induction of apoptosis or necrosis in ID8-T cells treated with sCXCR4-A (10 μg/mL), OVV, or OVV-CXCR4-A (MOI = 1) was determined by staining with Annexin V- fluorescein isothiocyanate (FITC) and LIVE/DEAD fixable violet (Thermo Fisher Scientific) according to the manufacturer’s instructions. Phenotypic analysis of tumor-infiltrating myeloid cells and T cells was performed on single-cell suspensions prepared from peritoneal fluid collected 8 days after completion of the treatments. All antibodies were purchased from BD Pharmingen (San Jose, CA, USA), BD Biosciences (San Jose, CA, USA), and BioLegend, as detailed in Table S1. The phycoerythrin (PE)-labeled H-2D^b^/RMFPNAPYL tetramer WT1 and PE-labeled H2-K^b^/TSYKFESV vaccinia virus-specific tetramer B8R were obtained from the MHC Tetramer Production Facility (Baylor College of Medicine, Houston, TX, USA). Percentages of CD4^+^ T cells expressing Foxp3 were determined by intracellular staining using the BD Pharmingen Transcription Factor Buffer Set (BD Biosciences) according to the manufacturer’s protocol. For tetramer analysis, lymphocytes were also gated on cells that were negative for CD11b expression. Background staining was assessed using isotype control antibodies. Before specific antibody staining, cells were incubated with Fc blocker (anti-CD16/CD32 mAb) for 10 min and analyzed on the LRS II flow cytometer (BD Biosciences). Data analysis was performed using WinList 3D 7.1 (Verity Software House, Topsham, ME, USA).

### Statistical Analysis

All statistical analyses were performed using GraphPad Prism 6 (GraphPad, La Jolla, CA, USA). Unless otherwise noted, data are presented as mean ± S.D. combined with unpaired, two-tailed Student’s t test. Kaplan-Meier survival plots were prepared, and median survival times were determined for tumor-challenged groups of mice. Statistical differences in survival across groups were assessed using the log rank Mantel-Cox method. The threshold for statistical significance was set to p < 0.05.

## Author Contributions

A.M., M.P.K., M.G., and M.A.G. performed the experiments. M.O., K.O.O., A.J., and H.R. designed the experiments and performed data analysis and manuscript review. D.K. designed the experiments and analyzed and interpreted the data. M.P.K. and D.K. drafted the manuscript.

## Conflicts of Interest

The authors declare no competing interests.

## Supplemental Information

Document S1. Figures S1–S4 and Table S1

Document S2. Article plus Supplemental Information
