# Supplementary material for: Recruitment of Intratumoral CD103+ Dendritic Cells by a CXCR4 Antagonist-Armed Virotherapy Enhances Antitumor Immunity
Source: Mol Ther Oncolytics. 2019 Jul 3;14:233–45. doi: 10.1016/j.omto.2019.06.003 (PMC6667789; doi:10.1016/j.omto.2019.06.003)
Supplement: Document S1. Figures S1–S4 and Table S1 [file mmc1.pdf]

OMTO, Volume *14*

Supplemental Information

Recruitment of Intratumoral CD103$^+$ Dendritic Cells by a CXCR4 Antagonist-Armed Virotherapy Enhances Antitumor Immunity

Anna Mistarz, Marcin P. Komorowski, Matthew A. Graczyk, Margaret Gil, Aimin Jiang, Mateusz Opyrchal, Hanna Rokita, Kunle O. Odunsi, and Danuta Kozbor

SUPPLEMENTAL FIGURES

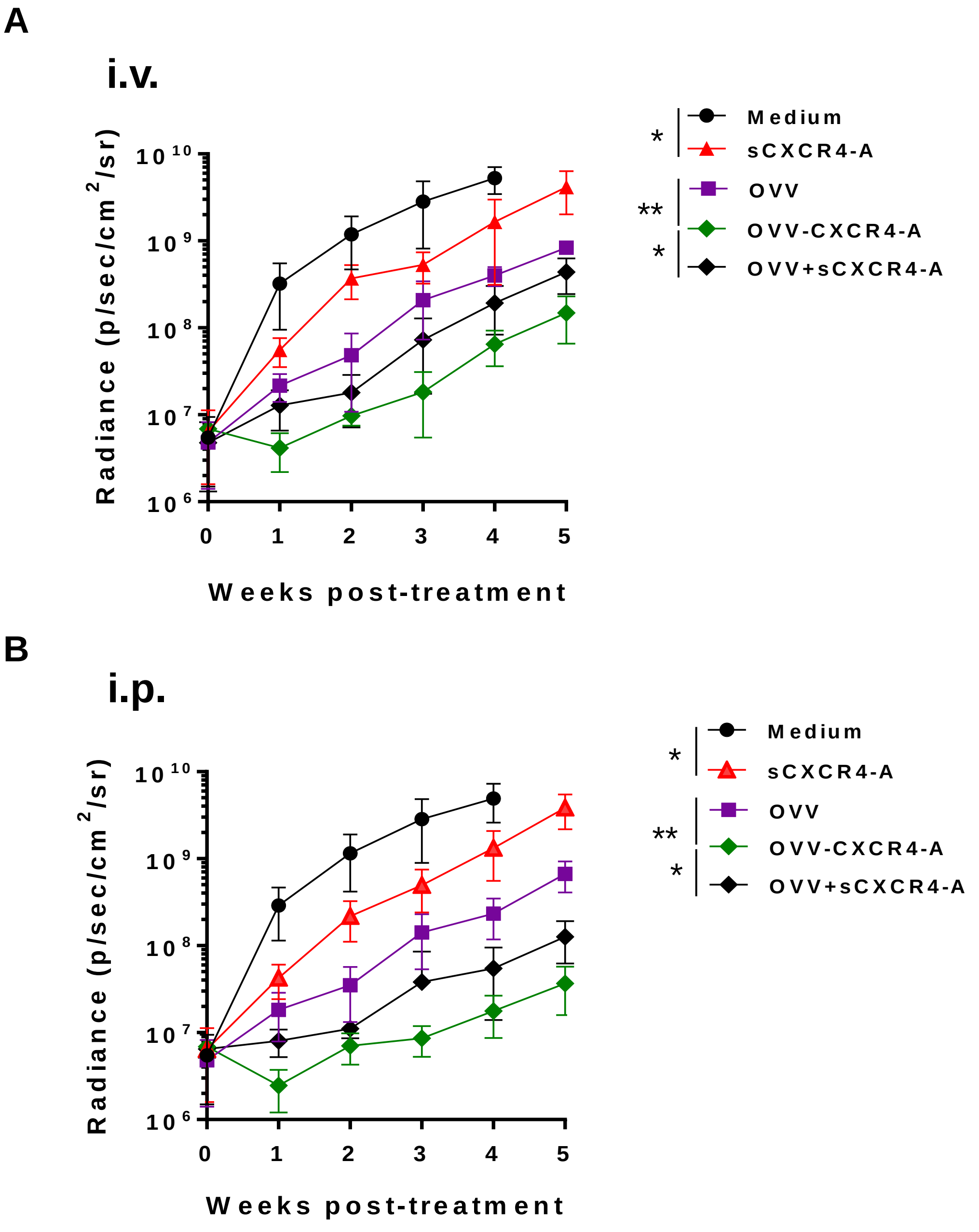

Figure S1. Local delivery of CXCR4-A is more effective at inhibiting tumor growth than systemic treatment. (**A, B**) C57BL/6 female mice (*n* = 6 - 10 per group) were challenged i.p. with $3x10^5$ ID8-T tumor cells. The tumor-bearing mice were treated with sCXCR4-A protein (10 mg/injection for 7 days), OVV, or OVV-CXCR4-A (10^8 PFU) injected i.v. (**A**) or i.p. (**B**) 10 days after tumor challenge. Control mice were treated with RPMI-1640 medium. Tumor progression was monitored by bioluminescence. Data points represent mean ± SD. **P* < 0.05, ***P* < 0.01.

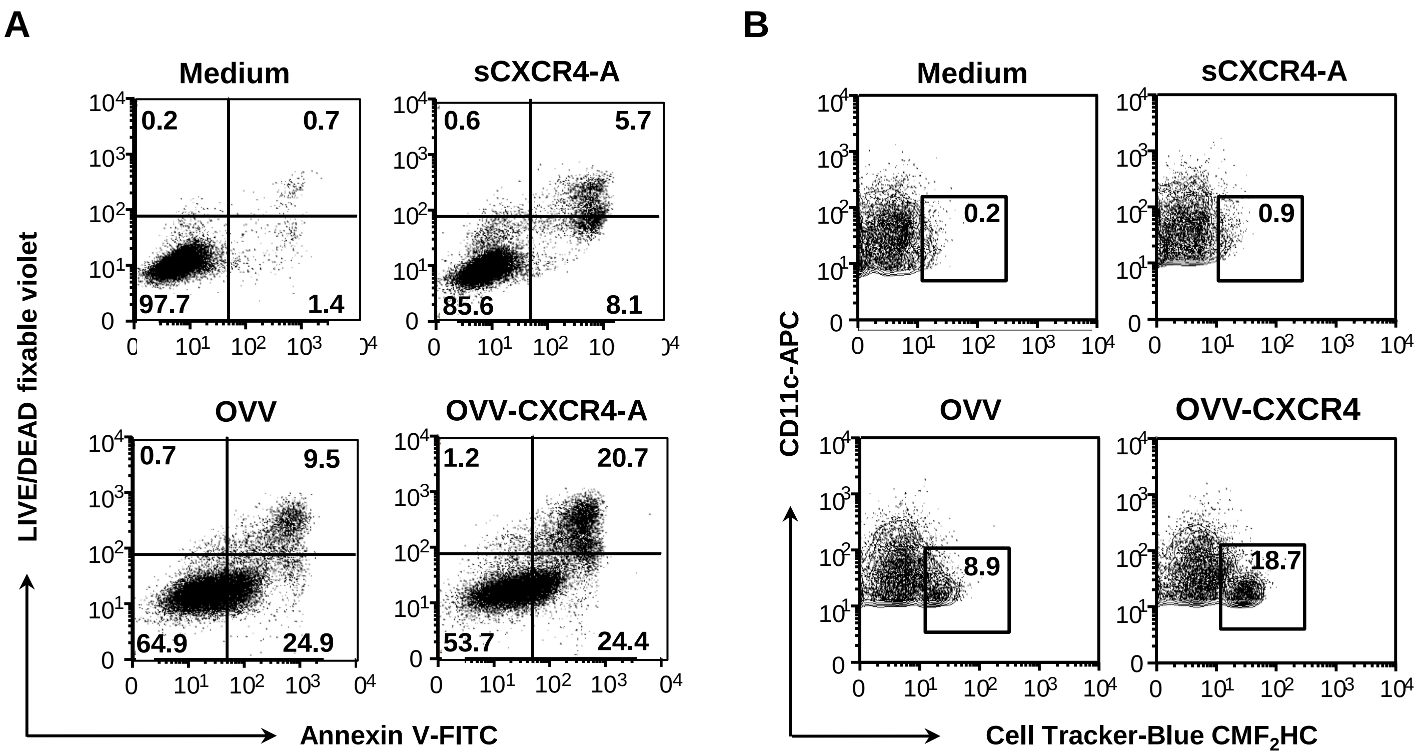

Figure S2. The CXCR4-A armed-virotherapy induces apoptosis associated with phagocytosis of tumor cell debris by DCs and delays tumor growth after FL-mediated expansion of CD103$^+$ DCs. (**A**) Cell death of ID8-T tumor cells treated with medium, sCXCR4-A (10 mg/ml), OVV or OVV-CXCR4-A (MOI = 1) was determined by staining with Annexin V-FITC and LIVE/DEAD fixable violet to measure the induction of early apoptosis (Annexin V$^+$/LIVE/DEAD fixable violet$^-$) and late apoptosis/necrosis (Annexin V$^{+/-}$/LIVE/DEAD fixable violet$^+$) by flow cytometry 24 h later. One representative experiment out of three performed is shown. (**B**) Phagocytosis of cell-tracker-blue CMF_2HC-labeled ID8-T tumor cells treated with medium, sCXCR4-A, OVV, or OVV-CXCR4-A by BM-derived, CD11c$^+$ DCs. Tumor cells were labeled with tracker-blue CMF_2HC before treatment and, after inactivating the virus, co-cultured with BM-derived DCs (1:1 ratio) for 12 h followed by staining with CD11c-APC antibody and flow cytometry analysis of double-positive cells. The percentages of CD11c-expressing DCs taking up tumor cell debris are indicated. One representative experiment of three independent experiments performed is shown.

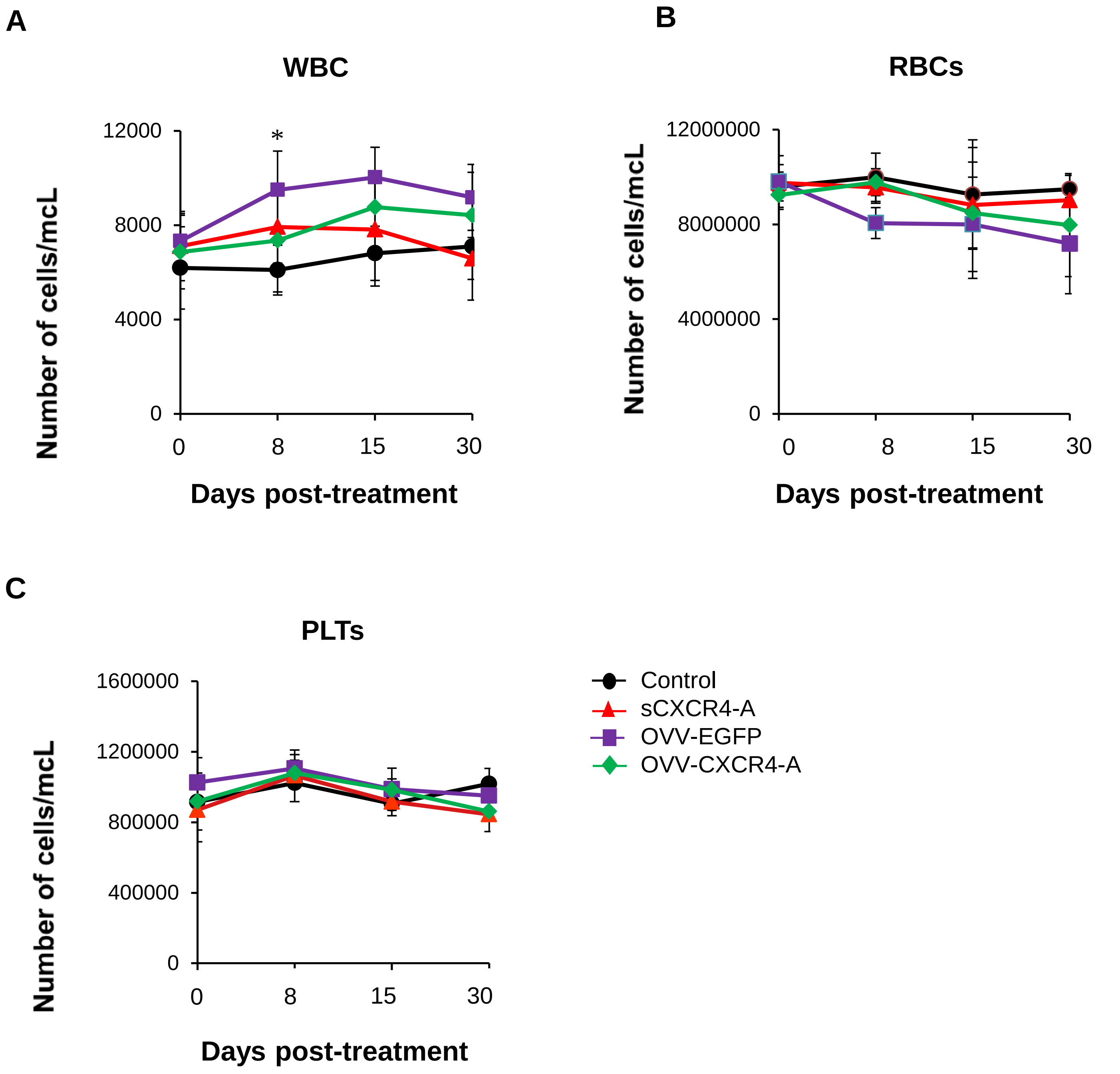

Figure S3. Cell blood count (CBC) after treatment with CXCR4 antagonist-armed OVV or sCXCR4-A delivered i.v. to ID8-T tumor-bearing mice. Mice (*n* = 5) were bled from the retro-orbital sinus to obtain complete counts of WBCs (**A**), RBCs (**B**), and PLTs (**C**) before treatment and on days 8, 15 and 30 after treatment initiation. The numbers of WBCs, RBCs, and PLTs in the heparinized blood samples were determined using IDEXX ProCyte Dx Hematology analyzer (IDEXX Laboratories, Inc., Westbrook, ME). Data are presented as the mean ± SD of five mice per group. *$P < 0.05$.

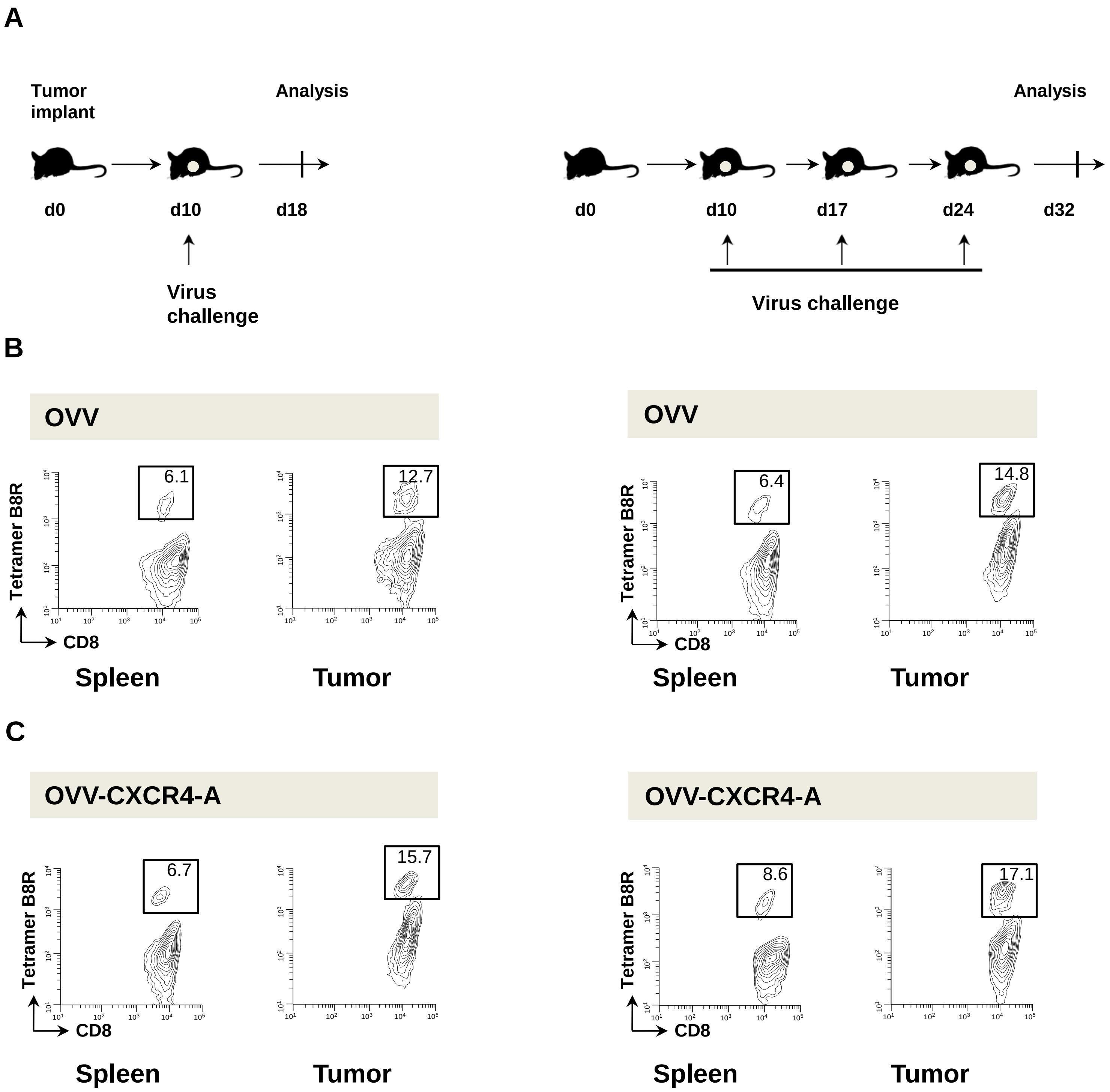

Figure S4. The effect of a single or multiple oncolytic virotherapy treatment on accumulation of B8R tetramer$^+$CD8$^+$ T cells in spleen and peritoneal cavities of ID8-T tumor-bearing syngeneic mice. (**A**) Graphical time line of the treatment scheme in ID8-T tumor-bearing mice. C57BL/6 mice were injected i.p. with 3 x 10^5 ID8-T cells. Treatment with OVV or OVV-CXCR4-A (10^8 PFU delivered i.p.) was initiated 10 days later. An additional group of mice was injected three times with the oncolytic viruses in a weekly intervals. The accumulation of B8R tetramer$^+$CD8$^+$ T cells in spleen and peritoneal cavities after treatment with OVV (**B**) or OVV-CXCR4-A (**C**) was analyzed by flow cytometry 8 days after treatment completion. One representative experiment of three independent experiments performed is shown.

SUPPLEMENTAL TABLE

Antibody	Clone	Source
CD45-V450	30-F11	BD Pharmingen
CD45-PerCP-Cy5.5	30-F11	BD Pharmingen
CD4-PE	GK1.5	BD Pharmingen
CD8a-BV786	53-6.7	BD Pharmingen
CD11b-BV786	M1/70	BD Pharmingen
Ly6G-PE	1A8	BD Pharmingen
Ly6C-FITC	AL-21	BD Pharmingen
CD11c-APC	HL3	BD Pharmingen
CD103-BV421	M290	BD Pharmingen
CD24-Alexa Fluor 700	M1/69	BD Pharmingen
CD25-FITC	PC61	BioLegend
I-A/I-E-BV605	M5/114.15.2	Biolegend
F4/80-PE	BM8	BioLegend
Foxp3-Alexa Fluor 647	MF-14	BioLegend
CD45R/B220	RA3-6B2	BD Biosciences
CD44-PerCP-Cy5.5	IM7	BD Pharmingen
CD62L-PE-Cy7	MEL-14	BD Pharmingen

Table S1. Monoclonal antibodies used in flow cytometry analysis.
